# ExoMars Raman Laser Spectrometer (RLS): development of chemometric tools to classify ultramafic igneous rocks on Mars

**DOI:** 10.1038/s41598-020-73846-y

**Published:** 2020-10-12

**Authors:** Marco Veneranda, Guillermo Lopez-Reyes, Jose Antonio Manrique-Martinez, Aurelio Sanz-Arranz, Emmanuel Lalla, Menelaos Konstantinidis, Andoni Moral, Jesús Medina, Fernando Rull

**Affiliations:** 1 Department of Condensed Matter Physics, Crystallography and Mineralogy, University of Valladolid, Ave. Francisco Vallés, 8, 47151 Boecillo, Spain; 2 Centre for Research in Earth and Space Science, York University, Toronto, Canada; 3 Department of Space Programs, National Institute of Aerospace Technology (INTA), Madrid, Spain

**Keywords:** Mineralogy, Characterization and analytical techniques, Raman spectroscopy

## Abstract

This work aims to evaluate whether the multi-point analysis the ExoMars Raman Laser Spectrometer (RLS) will perform on powdered samples could serve to classify ultramafic rocks on Mars. To do so, the RLS ExoMars Simulator was used to study terrestrial analogues of Martian peridotites and pyroxenites by applying the operational constraints of the Raman spectrometer onboard the *Rosalind Franklin* rover. Besides qualitative analysis, RLS-dedicated calibration curves have been built to estimate the relative content of olivine and pyroxenes in the samples. These semi-quantitative results, combined with a rough estimate of the concentration ratio between clino- and ortho-pyroxene mineral phases, were used to classify the terrestrial analogues. XRD data were finally employed as reference to validate Raman results. As this preliminary work suggests, ultramafic rocks on Mars could be effectively classified through the chemometric analysis of RLS data sets. After optimization, the proposed chemometric tools could be applied to the study of the volcanic geological areas detected at the ExoMars landing site (Oxia Planum), whose mineralogical composition and geological evolution have not been fully understood.

## Introduction

The ESA *ExoMars* rover mission aims to search for past/present life traces on Mars and to investigate the geochemical and environmental evolution of the planet^1,2^.

To fulfill these objectives the *Rosalind Franklin* rover will be equipped with a drill that reaches the up to 2 m in depth, thus collecting geological samples that have been sheltered from UV radiation and external environmental weathering processes^1^. Once collected, the rover Sample Preparation and Distribution System (SPDS) will crush the samples and deliver the powdered material to the analytical laboratory. Here, profiting from the combined analysis potential of the ExoMars rover^3^, the Raman Laser Spectrometer (RLS)^4^ and MicrOmega^5^ spectroscopic systems will investigate the mineralogical composition of the powders, which is a step of critical importance in the selection of the optimal scientific targets to be analyzed by the Mars Organic Molecule Analyzer (MOMA)^6^.

Among the mentioned instruments, RLS is the first Raman spectrometer to be qualified for space exploration missions^4^. The instrument will analyze between 20 and 39 spots per sample, depending on the available time and resources during operation. Having this in mind, it is of primary importance to carry out all the preparatory studies necessary to develop dedicated chemometric tools that can help maximizing the scientific outcome of Raman data sets (understood as the totality of spectra gathered from the sample). To this end, the RLS team developed the so-called RLS ExoMars Simulator, a laboratory spectrometer that collects spectra qualitatively comparable to those the RLS flight model (FM) will gather on Mars, while avoiding the technical-logistic limitations imposed by the management of instrumentation developed for space exploration^7^.

As demonstrated in preliminary studies based on the characterization of terrestrial analogs proceeding from the Planetary Terrestrial Analogues Library (PTAL) collection^8^, the RLS is able to effectively unveil the mineralogical complexity of heterogeneous geological samples, even achieving the detection of minor and trace phases^9^. Beyond the qualitative analysis of Raman spectra, further researches prove that the relative concentration ratio between major phases of sample mixtures could be determined through the chemometric analysis of RLS data sets^7^. Despite being preliminary results obtained under ideal conditions (mixture of analytically pure mineral phases exhibiting strong Raman scattering), the promising outcome encouraged the RLS science team to evaluate the possibility of using RLS data sets to extrapolate semi-quantification information from Martian rocks and soils.

Among the numerous potential applications, the semi-quantitative analysis of Martian samples having a low degree of complexity (in terms mineralogical diversity) could find a reliable use in the study of the igneous rocks that, according to remote data obtained from orbit^10,11^, cover more than 10% the landing site of the *ExoMars* rover (Oxia Planum). For example, it could be also used to correctly classify the olivine-rich rocks detected at the southern part of the ellipse, which indicates the presence of ultramafic geological units (dunite/peridotite) in the region. In this regard, the detailed analysis of CRISM, THEMIS and HiRISE high-resolution data from selected craters displaying unaltered central uplift structures, suggests that the Martian crust presents ultramafic igneous rocks dominated by olivine and pyroxene phases^12^. This inference about Martian crustal composition fits with the results obtained from the laboratory study of SNC Martian meteorites. Being mainly composed of olivine (≥ 90 vol%), with minor pyroxene, feldspar and chromite phases, Chassigny meteorites such as NWA 8694^13,14^ and NWA 2737^15,16^ are classified as dunite. Within the shergottite group, the lherzolitic variety (including Y984028^17^, LEW88516^18^ and ALH77005^18^, among others) are mainly composed of olivine (40–60 vol%) and clinopyroxene (mixture of pigeonite and augite, between 15 and 60 vol%) with minor chromite crystals^19,20^. Compared to these, nakhlites (e.g. Lafayette^21^, Nakhla^22^ and NWA817^23^) are mostly composed of monoclinic phases (augite, ≥ 75 wt%), while olivine is between 3 and 17 vol%^24^. Finally, Allan Hills 84,001 is mostly composed of orthorhombic pyroxene Fs_27.3_Wo_3.3_En_69.4_ (97 vol%, olivine concentration below 1 vol%)^25^, this being the only meteorite found to date belonging to the orthopyroxenite group. As described elsewhere^26,27^, by correlating the elemental and mineralogical composition of SNC meteorites with their formation ages, several patterns can be found that reflect an historical evolution of the Martian mantle—crust system.

Recognizing the scientific relevance of comparing the information extrapolated from Martian meteorites with the Raman data collected in situ from potentially-unaltered rocks, this work aims to find out whether, by combining an on-ground qualitative and semi-quantitative analysis of RLS-Simulator data sets, it is possible to achieve a correct classification of ultramafic igneous rocks on Mars. To achieve this goal, the present analytical study has been organized as follows. First, a Raman-based qualitative study of pyroxenite and peridotite analogues was carried out. Olivine/pyroxene calibration curves were then created and used to perform a semi-quantification study of the selected samples. Qualitative and semi-quantitative results were finally used to properly classify the analyzed rocks.

Taking into account that (1) this study was carried out using the RLS ExoMars Simulator by faithfully reproducing the operational mode of the RLS instrument on Mars, and (2) the Raman-based classification of terrestrial analogues was validated by comparison with reference data gathered from a state-of-the-art laboratory XRD system, this work aims to provide a realistic assessment of the potential scientific outcome that could derive from a refined chemometric analysis of RLS data sets gathered on Mars.

## Materials and methods

### Terrestrial analogue selection

In this work, Terrestrial analogs of Martian peridotite and pyroxenite were selected and analyzed. Peridotites were collected in 2009 from the Svalbard islands (Norway) during the Arctic Mars Analogue Svalbard Expedition (AMASE) coordinated by NASA and ESA agencies^28^. Svalbard peridotitic rocks are well acknowledged for being optimal terrestrial analogues of Martian ultramafic geological units. In fact, there are numerous studies in which these materials have been used to test space-derived analytical instruments^29^, and to simulate mineral weathering processes potentially occurring on Mars^30^. In detail, the analogues considered in this work (DUN1, DUN2 and DUN3) are coarsely crystalline rock of green color that, according to preliminary petrographic evaluations, mainly consists of olivine and pyroxene minerals.

Pyroxenite terrestrial analogues (named PYR1, PYR2 and PYR3) are part of the sample collection from the ERICA research group and were collected in northern Norway. Although the exact sampling area is unknown, many localities from northern Norway are widely acknowledged for being optimal terrestrial analogs of Martian geological contexts^8^. The selected coarse materials are dark in color and, based on microscopic observations, are mainly composed of pyroxene crystals with minor but varied amounts of olivine grains.

### Sample preparation

#### Terrestrial analogues

To accurately simulate the operational constraints of the RLS flight model, terrestrial analogues were pre-treated to reproduce the granulometric distribution of the SPDS Crusher. According to the ExoMars mission requirements, the average grain size of the crushed sub-soil samples must be ≈ 250 μm, with 90 wt% of the granulometry within 50 and 500 μm. Thus, terrestrial analogs were ground using a Planetary Mono Mill PULVERISETTE 6 (Fritsh) and sieved. The aliquots were then mixed by replicating the particle size distribution produced by the SPDS Crushing Station. For each analogue, an additional fragment was further milled to obtain fine powders with the grain sizes necessary to perform optimal XRD analyses (below 150 µm).

#### Mineral mixtures

A reliable semi-quantification study of the abovementioned ultramafic rocks passes through the use of external calibration curves that, in this work, were prepared by analyzing mixtures of olivine and pyroxene at different concentration ratios. For this purpose, pure and well characterized mineral phases were used. Although Martian olivine is rich in Fe (Fayalite end-member)^31–33^, these specimens are rarely found on Earth. For this reason, a certified forsterite (Mg-rich phase) from the Bureau of Analysed Samples LRD (reference code SX49-12) was used. On the other side, pure augite crystals from the Umba Valley region (Kenya) were selected as pyroxene proxy, this being one of the phases most frequently detected on Mars^34,35^. Furthermore, as explained in “Raman data treatment and analysis” section, the analytical method used for the calculation of the calibration curves is designed to be robust against the spectral variations expected from the different possible phases of the endmembers.

Olivine and pyroxene were manually milled using an agate mortar, sieved and mixed at different concentration ratios to obtain 11 reference samples (Table 1). As for the terrestrial analogues, the granulometric distribution of reference samples closely resembled the powders produced by the ExoMars Crushing station.

**Table 1:** Mineralogical composition of standard sample mixtures used for the external calibration procedure.

Sample ID	Pyroxene (wt%)	Olivine (wt%)
P0	0.00	100.00
P5	5.00	95.00
P10	9.93	90.07
P25	25.15	74.85
P37.5	37.00	63.00
P50	50.00	50.00
P62.5	69.49	30.51
P75	75.12	24.88
P90	89.40	10.60
P95	95.00	5.00
P100	100.00	0.00

### Analytical instruments

#### X ray diffractometry

A Discover D8 X-Ray Diffractometer (Bruker) was used to investigate the mineralogical composition of the terrestrial analogues. The instrument includes a Cu X-ray tube (wavelength 1.54 Å) as excitation source and a LynxEye detector. Fine-powdered rocks (granulometry ≤ 150 µm) were analyzed by setting a scan range between 5° and 70° 2θ, a step increment in 2θ of 0.01 and a count time of 0.5 s per step. Analysis of resulting diffractograms was performed with the XPowder 2004.04.71 software with PDF-2 (2010) and the American Mineralogist Crystal Structure Database crystallographic databases. A background correction for each diffractogram was achieved with the Splin-autoroller and polynomial tools available XPowder 2004.04.71 that allows for calculation of a background polynomial subtraction function^36^. XRD mineral quantification and theoretical density determination of the mixture were achieved by using the reference intensity ratio from pattern matching results with XPowder 2004.04.71^36^.

#### Raman spectroscopy

The Raman analysis of these ultramafic rocks was performed by means of the RLS ExoMars Simulator^7^, which is considered the most reliable laboratory spectrometer to effectively emulate the scientific outcome of the RLS system onboard the *Rosalind Franklin* rover^8,9,37^. The instrument includes a continuous laser source emitting at 532 nm, a high resolution TE Cooled CCD Array spectrometer, an optical head with a long WD objective of 50 × and a reply of the ExoMars sample holder. Range of analysis (70–4200 cm^−1^), working distance (≈ 15 mm), laser power output (20 mW), spot of analysis (≈ 50 µm), spectral resolution (6–10 cm^−1^) and signal to noise ratio of this instrument are closely resembling those of the RLS instrument. Software-wise, the RLS ExoMars Simulator integrates the same algorithms implemented by the RLS to autonomously operate on Mars, such as dark subtraction, fluorescence quenching and acquisition parameters calculation^38^. Data were acquired using a custom developed software based on LabVIEW 2013 (National Instruments).

Considering the RLS instrument soon operating on Mars will analyze a line of 20–39 spots per sample, 5 lines of 39 spots were run on each terrestrial analogue as well as on the 11 olivine/pyroxene mixtures. Qualitative and semi-quantitative information extrapolated from the 5 data sets were then compared in order to determine the reproducibility of the results.

### Raman data treatment and analysis

Prior to the qualitative data analysis, Raman spectra were submitted to baseline correction, elimination of cosmic rays and normalization. This task was performed by using the IDAT/SpectPro, a software developed by the RLS team to receive, decodify, calibrate and verify the telemetries generated by the RLS on Mars^39^. After treatment, Raman data sets gathered from olivine/pyroxene mineral mixtures were used to build dedicated calibration curves. Being this the first documented approach towards the potential use of RLS data sets to semi-quantify terrestrial analogues of Martian ultramafic rocks, authors chose to apply an univariate chemometric method which is, in turn, robust against the possible variations in the peak positions of the mixtures endmembers, making the method reliable for the quantification of olivine-pyroxene mixtures, irrespective of their specific mineral phase. As experimented elsewhere, calibration curves were generated by calculating the intensity ratio between the main peaks of the considered phases (in this case ≈ 855 and ≈ 660 cm^−1^ for olivine and pyroxene respectively) with respect to their total intensity^7^. For each mixture, a mean value was obtained for each line of spots by averaging the result of the respective spectra (see Eqs. 1, 2).

1 $$r_{oli} = \frac{1}{n}\cdot\mathop \sum \limits_{n} \frac{{I_{oli} }}{{I_{oli} + I_{pyr} }};$$

2 $$r_{pyr} = \frac{1}{n}\cdot\mathop \sum \limits_{n} \frac{{I_{pyr} }}{{I_{oli} + I_{pyr} }};$$

where r is the estimated proportion concentration indicator (related to the proportion through the calculated calibration curve), n is the number of spots per line and *I* is the peak intensity of the spectrum (without baseline) at a determined spot. The calibration curve uncertainty was estimated by calculating the standard deviation among the mean values of the 5 lines, with a confidence interval of 95% (± 2σ). All the calculations were performed using MATLAB R2019a.

An additional interpretation of pyroxene spectra was then performed to estimate the concentration ratio between monoclinic (clino-) and orthorhombic (ortho-) phases. As detailed in previous works^40,41^, the Raman signal that most clearly differentiates the two systems is emitted by the Si–O symmetric stretching vibration of the bridging O atoms (Si–O_br_, 650 ÷ 750 cm^−1^). Indeed, monoclinic minerals emit a single, sharp peak around 665 cm^−1^, while orthorhombic phases show a characteristic doublet (signal of medium intensity at 662 cm^−1^ together with a stronger peak around 680 cm^−1^. Having this in mind, the exact position of the Si–O_br_ peaks was interpreted as follows:

- If the main peak is below 667 cm^−1^, the spectrum is assigned to monoclinic minerals.
- If the main peak is above 675 cm^−1^, the spectrum is assigned to orthorhombic minerals.
- If the peak is detected between 667 and 675 cm^−1^, the spectrum is assigned to both systems.

The concentration ratio between clino- and ortho-pyroxene was roughly estimated by calculating, for each line of spots, the ratio between the number of spectra assigned to each system. The uncertainty was finally estimated by calculating two-times the standard deviation of the ratio extrapolated from each line of spots (95% confidence bounds).

## Results

### Raman-based qualitative analysis of terrestrial analogues

**Figure 1:**
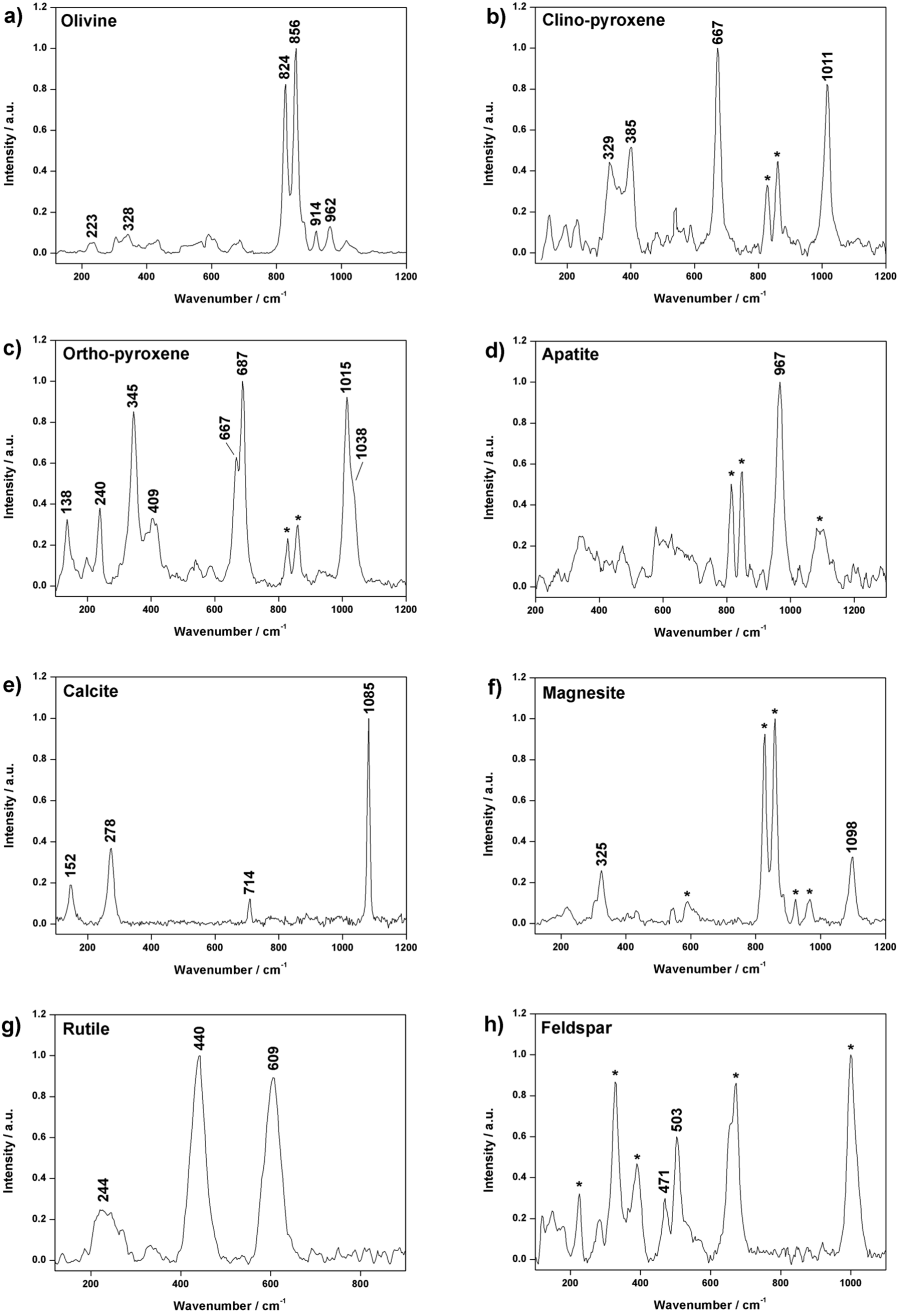
Characteristic RLS spectra gathered from the study of DUN and PYR samples. Peaks labelled with an asterisk proceeds from additional mineral phases. In detail, the displayed spectra were collected from sample DUN 1 (**a**, **e**), DUN 2 (**c**, **f**), DUN 3 (**g**), PYR 1 (**d**) and PYR 2 (**h**) and PYR 3 (**b**).

The characteristic doublet of olivine (824 and 856 cm^−1^) was found in most of DUN1 spectra. In some cases, the high SNR enabled the detection of additional secondary peaks at 591, 826, 921 and 965 cm^−1^ (Fig. 1a). As detailed in previous works, marked shifts in the position of Raman peaks may occur, being their position closely correlated to the elemental composition of the olivine grain. Even though similar displacement could be triggered by marked temperature variations, this issue will have a minimal influence on RLS spectra. Indeed, being analyzed inside the Pasteur payload, samples are expected to be stored at temperatures close to − 5 °C (thus, not far from laboratory conditions). Taking into account that olivine is a solid solution between forsterite (Fo, Mg_2_SiO_4_) and fayalite (Fa, Fe_2_SiO_4_) end-members, calibration curves were built to extrapolate the Fo/Fa ratio of olivine crystals by analyzing the position of their Raman peaks^42–44^. Considering the position of olivine peaks and applying the calibration curves presented by Mouri and Enami^43^, the composition of DUN 1 olivine grains can be described as Fo_95_Fa_05_. This value is consistent with the results provided in previous works that, through the elemental study of Svalbard peridotites, detected olivine grains with a composition ranging between Fo_90_Fa_10_ and Fo_91_Fa_09_^45,46^. In this regards, it must be underlined that some olivine spectra from DUN 1 displayed a peak shift displacement of 1 cm^−1^ (823 and 855 cm^−1^). However, the spectral resolution of the spectrometer (6–10 cm^−1^) does not allow to define with certainty whether the observed displacement was due to variations in the elemental composition of the mineral or to instrumental factors.

Beside olivine, the detection of intense peaks around 670 and 1010 cm^−1^ and secondary peaks in the spectral region below 400 cm^−1^ confirmed the presence of pyroxene as additional major phase. Knowing that variations in the spectral features of pyroxene can be used to distinguish monoclinic and orthorhombic phases^41^, the presence of two different minerals was deduced. On one hand, numerous spectra displayed peaks at 329, 385, 667 and 1011 cm^−1^, which perfectly match augite reference patterns (clino-pyroxene, (Ca,Mg,Fe)_2_(Si,Al)_2_O_6_, Fig. 1b). On the other hand, a lower number of spectra showed peaks at 138, 240, 345, 667, 687, 1015 and 1038 cm^−1^, fitting enstatite standards (ortho-pyroxene, MgSiO_3_, Fig. 1c).

At least one spectrum of calcite was detected on each line analyzed from sample DUN 1 (main peak at 152, 278, 714 and 1085 cm^−1^, see Fig. 1e). Calcium carbonate can be interpreted as product of metasomatism alteration that, as confirmed by detailed mineralogical studies of equivalent samples, generated from the interaction between mantle rocks and carbonatite fluids^47–49^. Together with calcite, apatite was also found as minor phase (Ca_5_(PO_4_)_3_, Main peak at 967 cm^−1^, see Fig. 1d). This mineral can also form under hydrothermal conditions and, as described in previous studies, it is found as mineral indicator of carbonatite metasomatism of mantle xenoliths^47–49^. This provides an additional evidence in support of the occurrence of this specific alteration mechanism in the analyzed analogues.

Sample DUN 2 presents a mineralogical composition similar to DUN 1. Once again, olivine was found to be the main mineral phase. However, the characteristic double peak was constantly detected at 823 and 855 cm^−1^, suggesting an elemental composition closer to the forsterite endmember (Fo_92_Fa_08_). Pyroxene was also detected as additional major phase. In this case, however, augite was only sporadically detected, suggesting the predominant presence of orthorhombic phases (enstatite). Rock alteration by metasomatism carbonatites was verified by the detection of calcite and magnesite (MgCO_3_, main peaks at 325 and 1098 cm^−1^, Fig. 1f). The joint presence of the two carbonates on Svalbard ultramafic rocks fits with the results presented in previous works^50,51^.

Sample DUN 3 is mainly composed of olivine (Fo_92_Fa_08_) and pyroxenes. In this case, the relationship between monoclinic and orthorhombic phases appeared to be similar to sample DUN 2, being enstatite the most commonly detected mineral. Rutile (TiO_2_) was additionally found as accessory phase. The main peaks of this titanium oxide polymorph (244, 440 and 609 cm^−1^, see Fig. 1g) were clearly detected in all data sets. Concerning metasomatism alteration products, apatite was also observed, while the presence of calcite is doubtful (a very weak signal was sporadically observed between 1080 and 1090 cm^−1^).

Under a qualitative point of view, PYR1, PYR2 and PYR3 show very similar compositions. They are mostly composed of pyroxene phases, with olivine as additional mineral. As for the case of DUN samples, PYR analogues are a mixture of orthorhombic and monoclinic phases. From a preliminary observation, the majority of the spectra gathered from samples PYR1 and PYR3 display a double peak in the range between 650 and 700 cm^−1^, suggesting a high concentration of clino-pyroxene grains. On the contrary, the main mineral phase of sample PYR 2 has a monoclinic structure. In PYR samples, the main olivine peaks were constantly detected at 822 and 853 cm^−1^ (Fo_87_Fa_13_). According to the number of spectra collected from each analogue, it can be estimated that PYR1 and PYR3 samples has a similar olivine content, which is higher than PYR2. As represented in Fig. 1h, one spectrum (out of 196) of feldspar was detected on sample PYR2. However, this mineral is incompatible with ultramafic igneous rocks, thus it must be interpreted as a contamination occurred during sample collection and/or preparation. Beside feldspar, neither minor primary minerals nor alteration products were detected in the analyzed materials.

The overview of the detected mineral phases is provided in Table 2.

**Table 2:** Summary of mineral phases detected by means of RLS ExoMars Simulator.

Minerals	DUN 1	DUN 2	DUN 3	PYR 1	PYR 2	PYR 3
Olivine	X	X	X	X	X	X
Clino-pyroxene	X	X	X	X	X	X
Ortho-pyroxene	X	X	X	X	X	X
Feldspar					?	
Rutile			X			
Calcite	X	X	?			
Magnesite		X				
Apatite	X		X	X		

### Construction of olivine/pyroxene calibration curves

**Figure 2:**
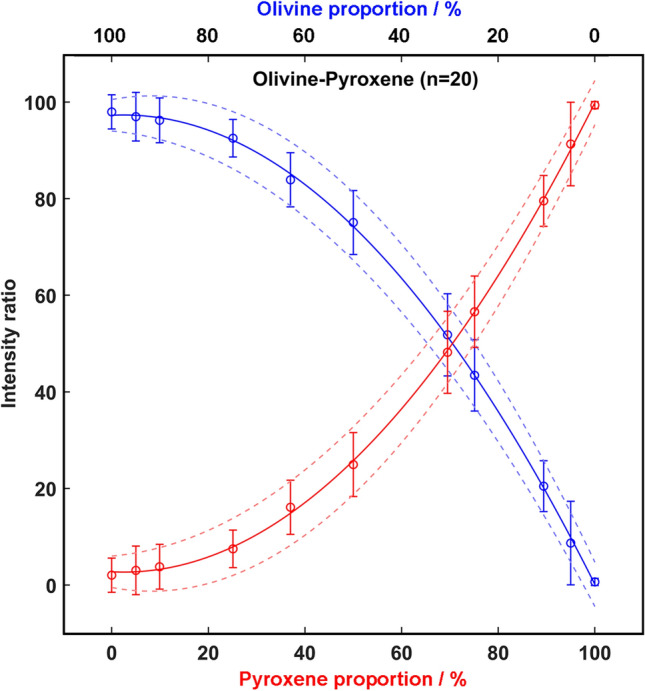
Pyroxene and olivine calibration curves for 20-point lines.

Laboratory samples, prepared by mixing olivine and pyroxene at controlled proportions, were used to build calibration curves by following the method described in “Raman data treatment and analysis” section. Following the method described elsewhere^7^, the intensity ratio between the main peaks of olivine and pyroxene (≈ 855 and 660 cm^−1^ respectively) was calculated for each spectrum. The relative concentration ratio was calculated by averaging the value obtained from each spectrum composing the line of spots. The uncertainty was finally estimated within 95% confidence bounds by calculating two-times the standard deviation of the proportions gathered from the 5 lines of spots. Knowing that (1) the number of analysis the RLS will perform on Martian samples will vary from 20 to 39, and (2) the reliability of the Raman-based semi-quantification method depends on the number of analyzed spectra, in this work two extreme scenarios were evaluated. In the first case, only the minimum number of spectra to be analyzed by RLS during nominal operation on Mars was considered (20). By plotting the calculated intensity ratio (Y axis) versus the real proportion (wt-%, X axis) of the samples listed in Table 1, the calibration curves represented in Fig. 2 were obtained.

Equations 3 and 4 describes the obtained calibration curves, which will be then used to estimate the relative concentration ratio between olivine and pyroxene of ultramafic rocks are:

3 $$r_{oli} \left( {20\, spots} \right) = - 0.0102 \cdot prop_{oli}^{2} + 0.0503 \cdot prop_{oli} + 97.2588;\quad R^{2} = 0.9996$$

4 $$r_{pyr} \left( {20\,spots} \right) = 0.0102 \cdot prop_{pyr}^{2} - 0.0503 \cdot prop_{oli} + 2.7412;\quad R^{2} = 0.9996$$

**Figure 3:**
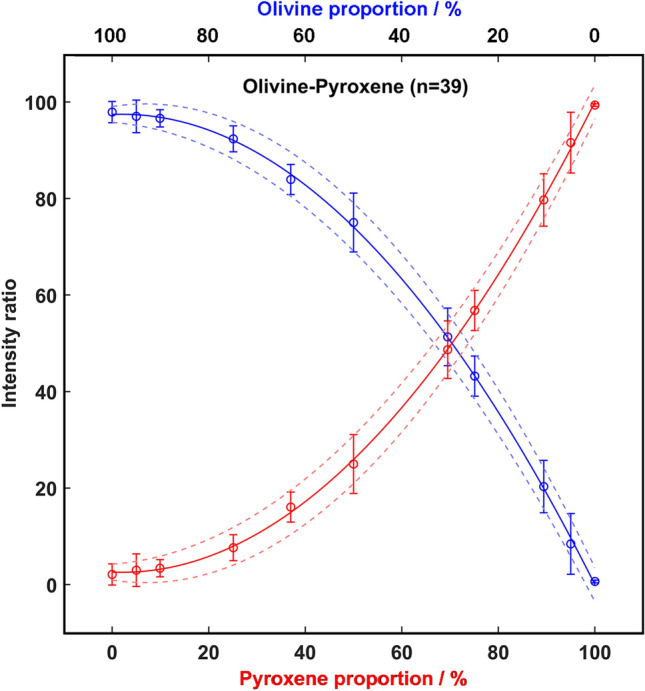
Pyroxene and olivine calibration curves for 39-point lines.

In the second case, the maximum number of spectra the RLS spectrometer will gather from Martian samples was considered (39). The obtained calibration curves are displayed in Fig. 3, while the relative equations (Eqs. 5, 6) are provided below.

5 $$r_{oli} \left( {39 \,spots} \right) = - 0.0102 \cdot prop_{oli}^{2} + 0.0410 \cdot prop_{oli} + 97.4123;\quad R^{2} = 0.9997$$

6 $$r_{pyr} \left( {39\,spots} \right) = 0.0102 \cdot prop_{pyr}^{2} - 0.0410 \cdot prop_{oli} + 2.5877;\quad R^{2} = 0.9997$$

**Figure 4:**
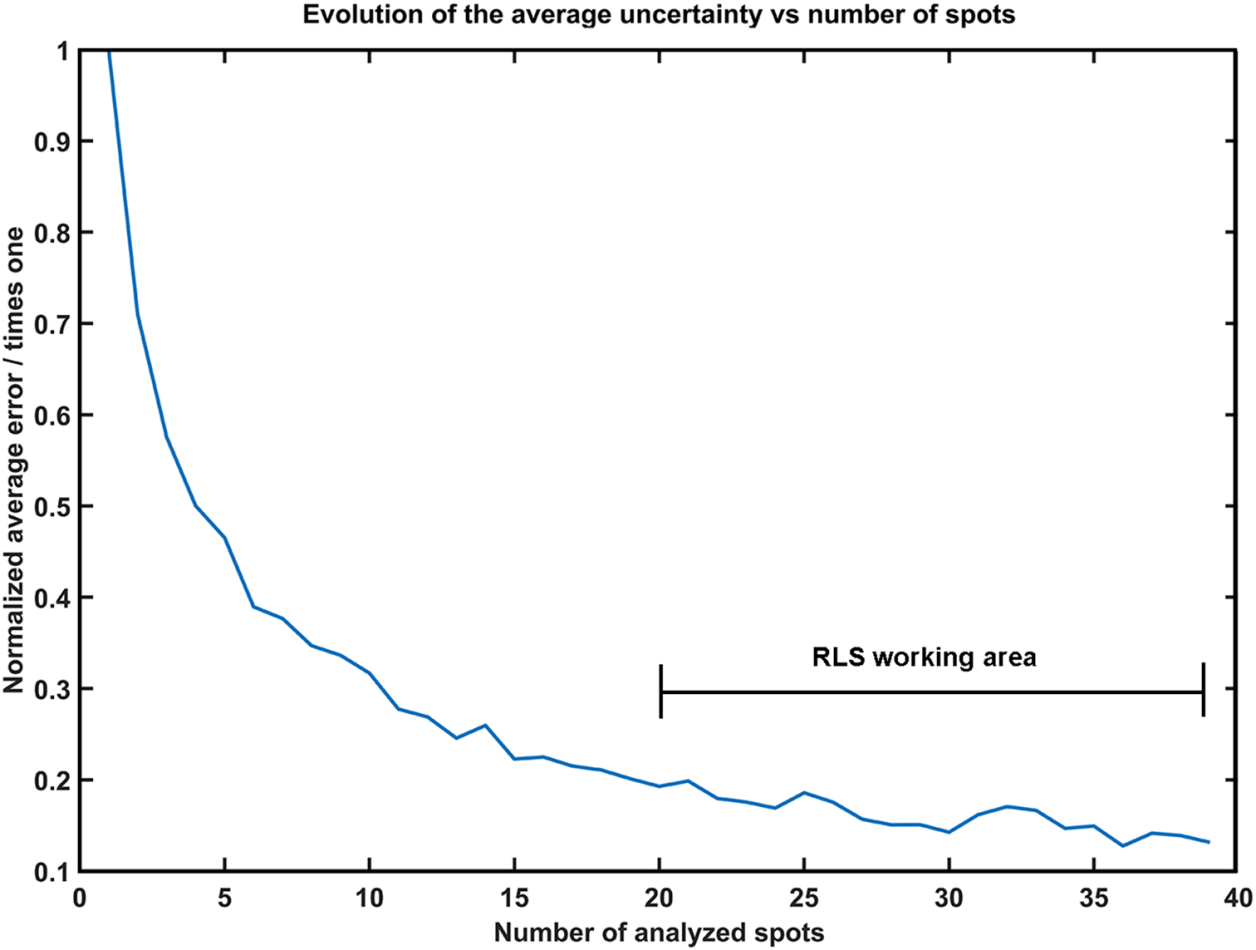
Prediction uncertainty as function of the number of analyzed spectra per line.

By comparing the two cases, 39-points curves present a similar tendency to the 20-points one, however, the uncertainty of the measure (calculated as ± 2σ) decreases from ± 5.4 to ± 3.7%. Considering the obtained results, the estimated proportion uncertainty of the calibration curves was evaluated for different numbers of spectra per line, from 1 to 39. As shown in Fig. 4, the proportion uncertainty markedly decreases starting from the analysis of 18 spectra. Thus, it was inferred that the relative concentration ratio between two main minerals composing Martian samples of low complexity (in terms of mineralogical heterogeneity) would be possible when working within the foreseen operational parameters of RLS (between 20 and 39 spots).

### Raman-based semi-quantitative analysis of ultramafic rocks

#### Concentration ratio between olivine and pyroxene

As in the case of mineral standard mixtures, ultramafic rocks were analyzed with the RLS ExoMars Simulator by collecting 5 lines of 39 spectra each. Though nominally this will not be the case for RLS while operating in Mars (the instrument will gather one line only), five lines were analyzed to provide a statistical reference of the expected uncertainty on the “problem” samples. The results gathered by applying the proposed univariate chemometric method are summarized in Table 3. As shown below, two values of uncertainty were calculated. The first one is extrapolated for each line from the calibration curve with 95% confidence bounds, while the second one is the standard deviation of the resulting proportions obtained from the five analyzed lines.

**Table 3:** Semi-quantitative results extrapolated from RLS ExoMars Simulator data (39-spots per line).

Sample	Line	Olivine (%)	Pyroxene (%)	Uncert from cal. curve (%)	Olivine (%)	Pyroxene (%)	Std between lines (%)
DUN 1	L1	74.4	25.6	3.9	75.7	24.3	7.1
	L2	70.2	29.8	4.2			
	L3	84.2	15.8	3.2			
	L4	67.9	32.1	4.3			
	L5	81.7	18.3	3.4			
DUN 2	L1	79.3	20.7	3.6	82.0	18.0	10.3
	L2	92.8	7.1	2.4			
	L3	82.8	17.2	3.3			
	L4	66.2	33.8	4.4			
	L5	88.9	11.1	2.8			
DUN 3	L1	85.3	14.7	3.1	74.7	25.3	6.6
	L2	75.3	24.7	3.9			
	L3	74.5	25.5	3.9			
	L4	68.8	31.2	4.3			
	L5	69.6	30.4	4.2			
PYR 1	L1	19.2	80.8	4.6	18.2	81.8	1.2
	L2	16.7	83.3	4.5			
	L3	19.4	80.6	4.6			
	L4	17.2	82.7	4.6			
	L5	18.5	81.5	4.6			
PYR 2	L1	4.2	95.8	3.8	4.5	95.5	1.6
	L2	3.2	96.8	3.7			
	L3	4.0	96.1	3.8			
	L4	7.3	92.7	4.0			
	L5	3.7	96.3	3.8			
PYR 3	L1	8.5	91.5	4.1	8.0	92.0	1.5
	L2	7.6	92.4	4.0			
	L3	7.3	92.7	4.0			
	L4	10.3	89.7	4.2			
	L5	6.3	93.7	4.0			

#### Concentration ratio between orthorhombic and monoclinic pyroxene

The relative concentration ratio between clino- and ortho- pyroxene was calculated for each sample by following the method described in “Raman data treatment and analysis” section. This proportion has a great scientific relevance, since it could be used to extrapolate information on the geological evolution of Mars. Indeed, considering that the concentration ratio between clino- and ortho-pyroxene in Martian rocks shown compositional trends with time^12,26,52^, this value can be used as mineralogical indicator to estimate the age of geological units. In this work, uncertainty values were obtained by calculating the standard deviation of the ratios deduced from the five lines of 39 spots. Results are summarized in Table 4.

**Table 4:** Ortho-/clino-pyroxene concentration ratio, extrapolated from RLS ExoMars Simulator data.

Sample	Line	Ortho-pyroxene (%)	Clino-pyroxene (%)	Std (%)	Sample	Line	Ortho-pyroxene (%)	Clino-pyroxene (%)	Std (%)
DUN 1	L1	33.3	66.7		PYR 1	L1	71.4	28.6	
	L2	25.0	75.0			L2	64.3	35.7	
	L3	40.0	60.0			L3	62.5	37.5	
	L4	16.7	83.3			L4	83.3	16.67	
	L5	33.3	66.7			L5	66.7	33.3	
Average		29.7	70.3	9.0	Average		69.6	30.4	8.4
DUN 2	L1	83.3	16.7		PYR 2	L1	80.9	19.1	
	L2	80.0	20.0			L2	89.2	10.8	
	L3	71.4	28.6			L3	94.9	5.1	
	L4	87.5	12.5			L4	86.8	13.2	
	L5	83.3	16.7			L5	97.5	2.5	
Average		81.1	18.9	6.0	Average		89.9	10.1	6.6
DUN 3	L1	54.5	45.5		PYR 3	L1	26.8	73.2	
	L2	77.8	22.2			L2	41.2	58.8	
	L3	70.0	30.0			L3	35.5	64.5	
	L4	75.0	25.0			L4	26.3	73.7	
	L5	66.7	33.3			L5	16.7	83.3	
Average		68.8	31.2	9.1	Average		29.3	70.7	9.4

Combining the results summarized on Tables 3 and 4, the mean concentration ratios of olivine, ortho- and clino-pyroxene was calculated for each sample (Table 5).

**Table 5:** Raman semi-quantitative data of the main mineral phases detected on DUN and PYR samples.

Sample	Olivine (%)	Ortho-pyroxene (%)	Clino-pyroxene (%)
DUN 1	75.7	7.2	17.1
DUN 2	82.0	14.6	3.4
DUN 3	74.7	17.4	7.9
PYR 1	18.2	57.0	24.8
PYR 2	4.5	28.0	67.5
PYR 3	8.0	82.7	9.3

### Raman-based classification of ultramafic rocks and result validation

From a qualitative point of view, the analyzed samples are mineralogically very similar. The six terrestrial analogues are mainly composed of olivine, clino-pyroxene and ortho-pyroxene, while rutile, apatite, calcite and magnesite were found to be additional minor phases of DUN samples. Considering the olivine-pyroxene proportions presented in “Raman-based semi-quantitative analysis of ultramafic rocks” section, the analyzed rocks can be divided in two groups. Due to a relative content of olivine between 74 and 82%. Rocks from Svalbard (DUN1-DUN3) can be classified as peridotites. On the contrary, PYR samples are dominated by pyroxene minerals, being the relative concentration of olivine ranging between 4 and 19%. According to the measured proportions, these samples can be classified as pyroxenite (olivine concentration below 60%). As detailed in “Concentration ratio between orthorhombic and monoclinic pyroxene” section, for a proper classification of ultramafic rocks the concentration ratio between clino- and ortho- pyroxene needs to be estimated. According to the chemometric analysis of RLS ExoMars Simulator data, DUN1 and PYR2 samples differ from the other analogues for having a higher concentration of monoclinic (over orthorhombic) phases.

**Figure 5:**
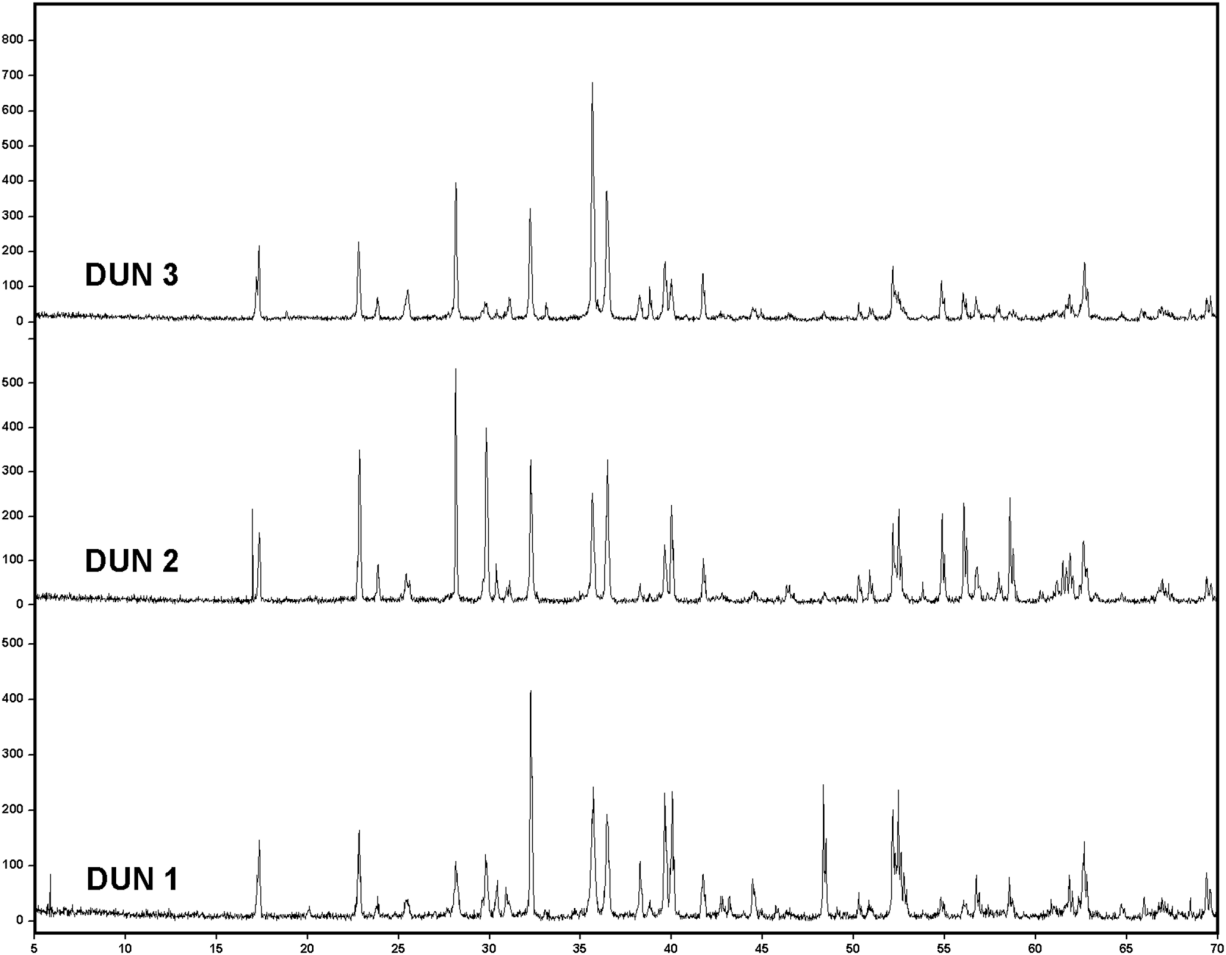
Diffractograms obtained from the analysis of DUN samples, highlighting the detection of olivine (Ol), ortho-pyroxene (Opx) and clino-pyroxene (Cpx).

**Figure 6:**
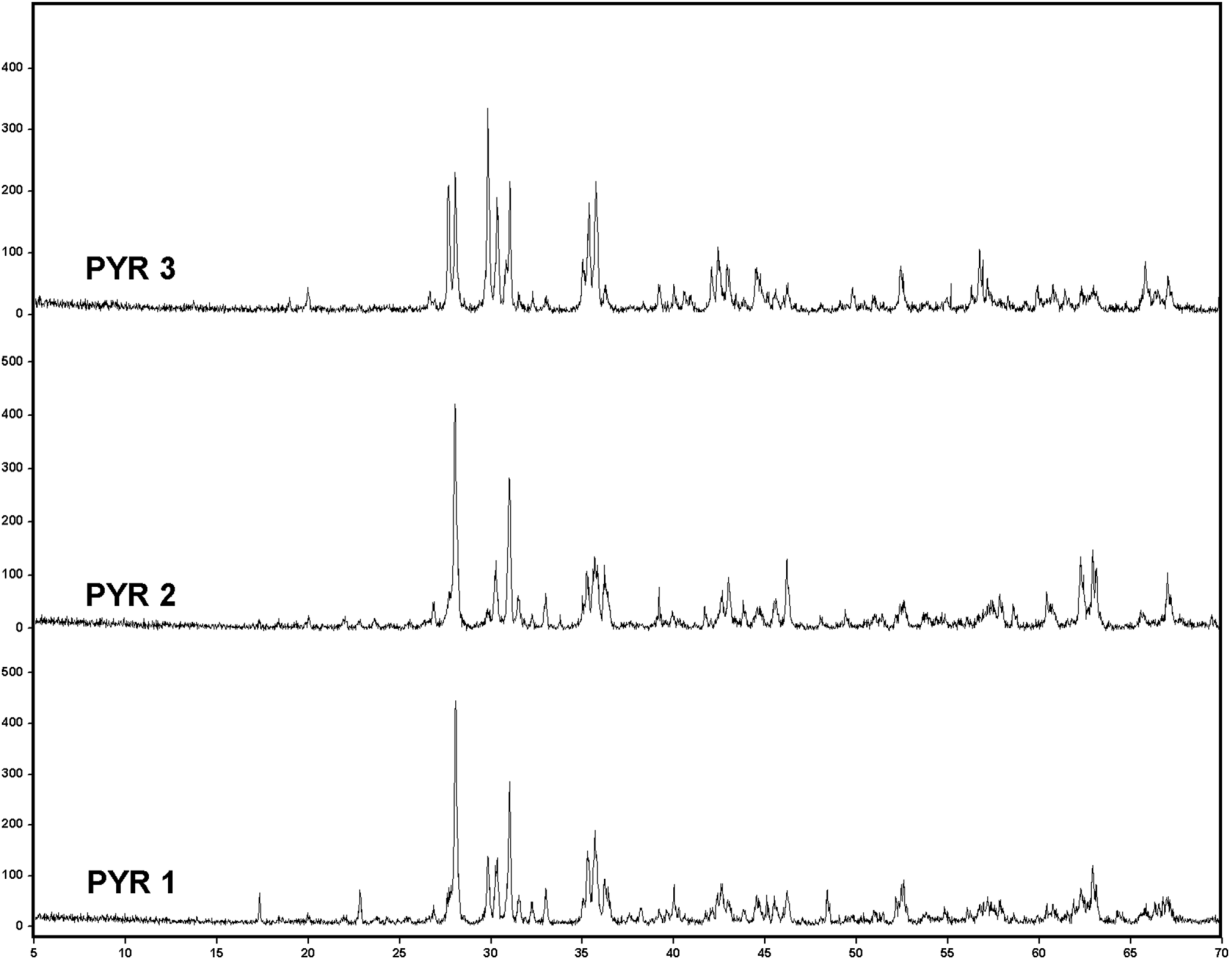
Diffractograms obtained from the analysis of PYR samples, highlighting the detection of olivine (Ol), ortho-pyroxene (Opx) and clino-pyroxene (Cpx).

Raman-based qualitative and semi-quantitative results were compared to those obtained from the use of a state-of-the-art XRD system. As displayed in Fig. 5, diffractograms from DUN samples reveal a mineralogy dominated by olivine with minor content of clino-pyroxene and ortho-pyroxene phases. On the contrary, PYR samples (Fig. 6) are mainly composed of pyroxene phases, while olivine is present in minor amounts.

Applying the quantification method described in “Analytical instruments” section, the values summarized in Table 6 were obtained. According to the model, the mean error estimated for the calculated concentrations is 3.0%.

**Table 6:** XRD semi-quantitative data of the main mineral phases detected on DUN and PYR samples.

Sample	Olivine (%)	Ortho-pyroxene (%)	Clino-pyroxene (%)
DUN 1	78.8	15.9	5.3
DUN 2	85.4	11.1	3.5
DUN 3	73.5	21.4	5.1
PYR 1	14.2	70.7	15.2
PYR 2	6.5	31.5	62
PYR 3	4.4	73.5	22.1

**Figure 7:**
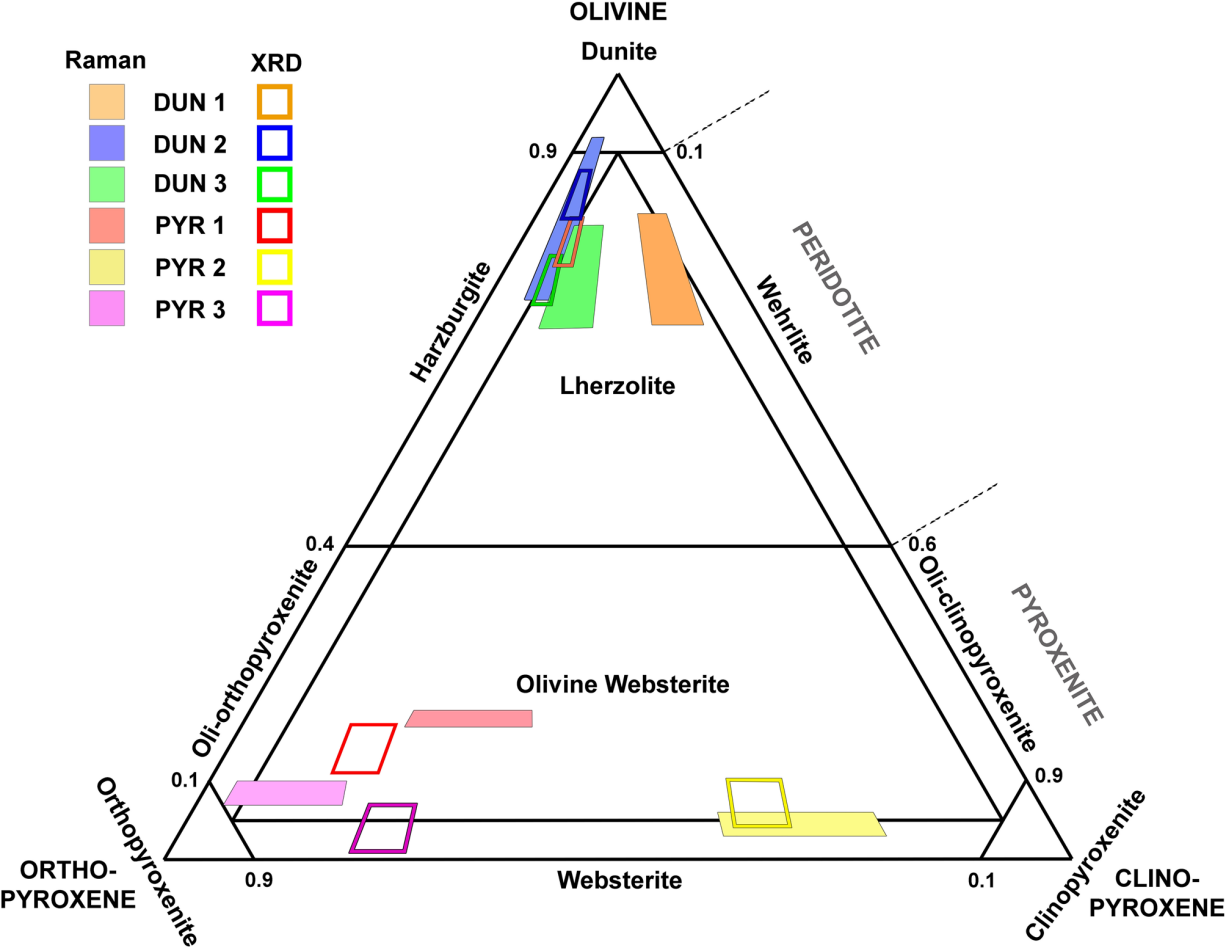
Ternary plot for sample classification.

Taking into account the olivine/pyroxene concentration ratio estimated for each sample, as well as the proportions between monoclinic and orthorhombic phases, XRD and Raman results were plotted in a ternary graph (olivine, clino-pyroxene and ortho-pyroxene as end-members). As shown in Fig. 7, the classification areas identified by the two techniques for samples DUN2, DUN3 and PYR2 are partially overlapped, thus proving the good agreement between Raman and XRD results. With regards to the other analogues, the olivine/pyroxene ratio extrapolated for the two techniques are also very similar, while the estimated ratio between orthorhombic and monoclinic phases is slightly different. Indeed, Raman results seem to underestimate the content of ortho-pyroxene in samples PYR1 and DUN1, and to overestimate it in sample PYR3.

Using diffractometric results as reference, the classification goodness of the proposed method was calculated by measuring the Euclidean distance between the mean concentration ratios estimated for each sample by XRD and Raman.

As shown in Fig. 7, DUN2 sample was classified as harzburgite by both semi-quantification methods. The overlap between XRD and Raman classification areas fit with the small Euclidian distance between their mean values (4.9). Even though both XRD and Raman instruments classified DUN1 and DUN3 as lherzolite, it must be underlined that the Euclidean distance measured from the two samples was 15.0 and 5.0 respectively. Relating this difference to the values provided in Tables 5 and 6, it can be inferred that the proposed Raman-based method underestimated the relative concentration of orthorhombic pyroxene (over monoclinic) on sample DUN1. PYR1 is the sample providing the greatest Euclidean distance (17.2) between XRD and Raman mean values (again, ortho-pyroxene concentration was underestimated by Raman). In spite of that, the analogue was classified as olivine-websterite by both semi-quantification methods. XRD and Raman results from PYR2 fit quite well (Euclidean distance = 6.9): both methods identified a composition between olivine-websterite and pure websterite. To conclude, the Euclidean distance between the areas calculated for PYR3 (16.1) caused this sample to be classified as websterite or olivine-websterite (rich in orthorhombic phases) depending if XRD or Raman data are considered, respectively. In this case, the discrepancy was mostly due to the Raman underestimation of clino-pyroxene content.

## Conclusions

As proved by the comparison with reference diffractograms, the RLS ExoMars Simulator used by emulating the operational mode established for the RLS system soon operating on Mars (between 20 and 39 spot of analysis per sample) was able to successfully disclose the mineralogical complexity of the analysed terrestrial analogues.

Calibration curves, obtained by analyzing laboratory-prepared mineral mixtures, and tested on Martian analogues, confirmed that RLS can be used to estimate the relative concentration ratio of olivine and pyroxene on powdered rocks. By emulating the operational constraints of the RLS instrument, a calibration curve with a correlation coefficients (R^2^) equal to 0.9997 with an estimated uncertainty of ± 3.7% (confidence interval = 95%) was obtained. Calibration curves were then used to extrapolate the relative content of olivine and pyroxene from the analyzed terrestrial analogues. The obtained results, combined with a rough estimation of the proportion between monoclinic and orthorhombic phases, were used to classify the igneous rocks. As can be seen in the ternary graph displayed Fig. 7, Raman results fit quite well with reference XRD data, being PYR3 the only sample where Raman and XRD brought to slightly different classifications (olivine-websterite vs websterite).

As the preliminary data summarized in this work suggests, the RLS spectrometer onboard the *Rosalind Franklin* rover could be used to correctly classify ultramafic rocks on Mars. This kind of study can find application in the analysis of the igneous geological units detected at the landing site, which have been pointed as potential analytical targets of the ExoMars mission.

Despite the good results obtained in this work, the classification method can be optimized. Being aware that the proposed univariate method can lead to under- or over-estimate the relative content of mineral phases depending, for example, on their crystallinity (factor that affects intensity and width of Raman peaks), the aim is to optimize the semi-quantification method by using a multivariate analytical approach. Furthermore, knowing that the main Si–O_br_ vibration signals of monoclinic and orthorhombic pyroxene partially overlap, the estimation of their relative proportion can be refined by implementing data analysis process with algorithms for automated spectra deconvolution. Depending on the quality of the spectra, secondary peaks could be also taken into consideration for a correct discrimination between pyroxene phases. In this sense, the RLS team is planning a dedicated work that, through the RLS (spare model) analysis of monoclinic and orthorhombic pyroxene standards, seeks to determine the optimal spectral indicators to use for a reliable discrimination between the two mineral structures. Knowing that Raman-based semi-quantification studies could optimize the scientific outcome of the RLS on Mars, this work describes the first steps of the RLS science team towards the development of the necessary chemometric tools.
